# Pre-existing depression in patients with coronary artery disease undergoing percutaneous coronary intervention

**DOI:** 10.1038/s41598-021-87907-3

**Published:** 2021-04-21

**Authors:** Jangho Park, Sangwoo Park, Yong-Giun Kim, Soe Hee Ann, Hyun Woo Park, Jon Suh, Jae-Hyung Roh, Young-Rak Cho, Seungbong Han, Gyung-Min Park

**Affiliations:** 1 Department of Psychiatry, Ulsan University Hospital, University of Ulsan College of Medicine, Ulsan, Republic of Korea; 2 Department of Cardiology, Ulsan University Hospital, University of Ulsan College of Medicine, 877 Bangeojinsunhwando-ro, Dong-gu, Ulsan, 44033 Republic of Korea; 3 Department of Cardiology, Soon Chun Hyang University Hospital Bucheon, Bucheon, Republic of Korea; 4 Department of Cardiology, Chungnam National University Hospital, Chungnam National University School of Medicine, Daejeon, Republic of Korea; 5 Department of Cardiology, Dong-A University Hospital, Busan, Republic of Korea; 6 Department of Biostatistics, College of Medicine, Korea University, 73, Goryeodae-ro, Seongbuk-gu, Seoul, 02841 Republic of Korea

**Keywords:** Cardiology, Epidemiology, Outcomes research, Depression

## Abstract

The impact of pre-existing depression on mortality in individuals with established coronary artery disease (CAD) remains unclear. We evaluate the clinical implications of pre-existing depression in patients who underwent percutaneous coronary intervention (PCI). Based on National Health Insurance claims data in Korea, patients without a known history of CAD who underwent PCI between 2013 and 2017 were enrolled. The study population was divided into patients with angina (n = 50,256) or acute myocardial infarction (AMI; n = 40,049). The primary endpoint, defined as all-cause death, was compared between the non-depression and depression groups using propensity score matching analysis. After propensity score matching, there were 4262 and 2346 matched pairs of patients with angina and AMI, respectively. During the follow-up period, there was no significant difference in the incidence of all-cause death in the angina (hazard ratio [HR] of depression, 1.013; 95% confidence interval [CI] 0.893–1.151) and AMI (HR, 0.991; 95% CI 0.865–1.136) groups. However, angina patients less than 65 years of age with depression had higher all-cause mortality (HR, 1.769; 95% CI 1.240–2.525). In Korean patients undergoing PCI, pre-existing depression is not associated with poorer clinical outcomes. However, in younger patients with angina, depression is associated with higher all-cause mortality.

## Introduction

Coronary artery disease (CAD) has been the leading cause of death worldwide for many years^1^. In Korea, CAD ranks second for mortality of all single-organ diseases^2^. In patients with CAD, depressive symptoms are common with a prevalence of 20%^3^. Depression is more prevalent during index hospitalization and follow up^4^.

Depression affects approximately 350 million people worldwide and is estimated to be the leading cause of disease burden globally by 2030^5^. In a meta-analysis, major depressive disorder was significantly associated with cardiovascular disease, CAD, cerebrovascular disease, and congestive heart failure^6^. In a national epidemiologic survey, the presence of a lifetime major depressive episode increased the risk of CAD in adults aged 60 years and older^7^. Accordingly, depression is considered an independent risk factor for cardiovascular disease, including CAD.

Subsequent depression after CAD events is an unfavorable prognostic factor. Depression after myocardial infarction is independently associated with a 2–3-fold higher risk of subsequent cardiovascular events^8^. In 2014, a scientific statement from the American Heart Association evaluated 53 studies and concluded that depression after acute coronary syndrome should be recognized as a risk factor for adverse outcomes^9^. However, it is still unclear whether pre-existing depression before CAD events is associated with poorer clinical outcomes.

Therefore, through a nationwide cohort using National Health Insurance (NHI) claims data in South Korea, we sought to investigate (1) whether pre-existing depression affects all-cause mortality among patients with established CAD who underwent PCI and (2) whether baseline depression influences all-cause death according to age or clinical diagnosis of CAD.

## Results

### Study population and characteristics

**Figure 1:**
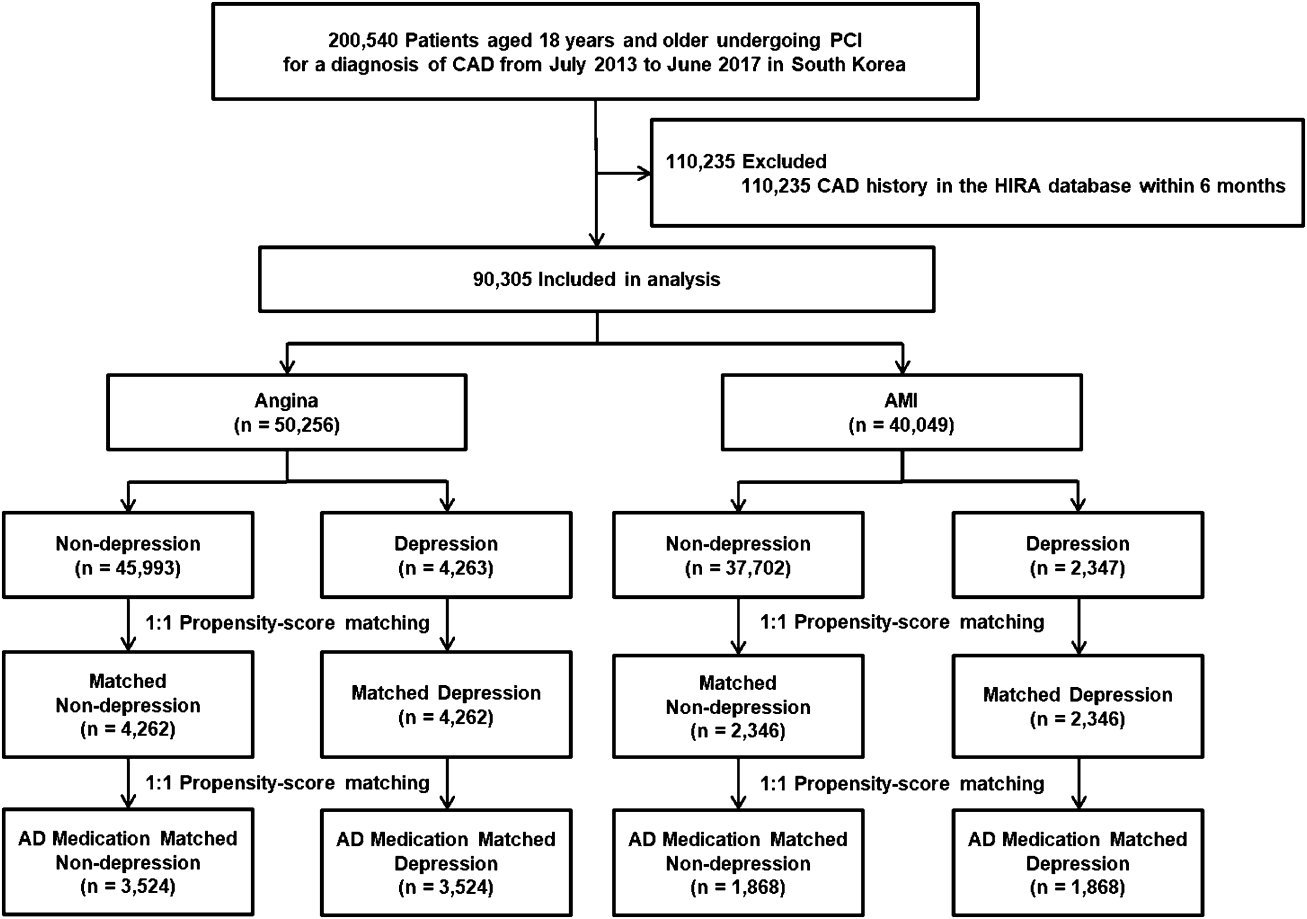
Overview of the study population. *AD* Antidepressants, *AMI* acute myocardial infarction, *CAD* coronary artery disease, *HIRA* Health Insurance Review and Assessment; *PCI* percutaneous coronary intervention.

Between July 2013 and June 2017, a total of 200,540 patients aged 18 years or older undergoing PCI for angina or AMI were identified from the claims database of the HIRA. Among them, 90,305 patients met the eligibility criteria and were selected as the study population. A total of 50,256 patients had a diagnosis of angina (angina group), and 40,049 patients were diagnosed with AMI (AMI group) as the first CAD event (Fig. 1). The mean age of participants was 64.6 ± 12.2 years, and 64,151 participants (71.0%) were male. The mean Charlson comorbidity index was 1.25 ± 1.40. During a median follow up of 2.2 years (interquartile range, 1.2–3.3), 7918 deaths (8.8%) were observed.

### Baseline characteristics of the participants according to depressive episode

**Figure 2:**
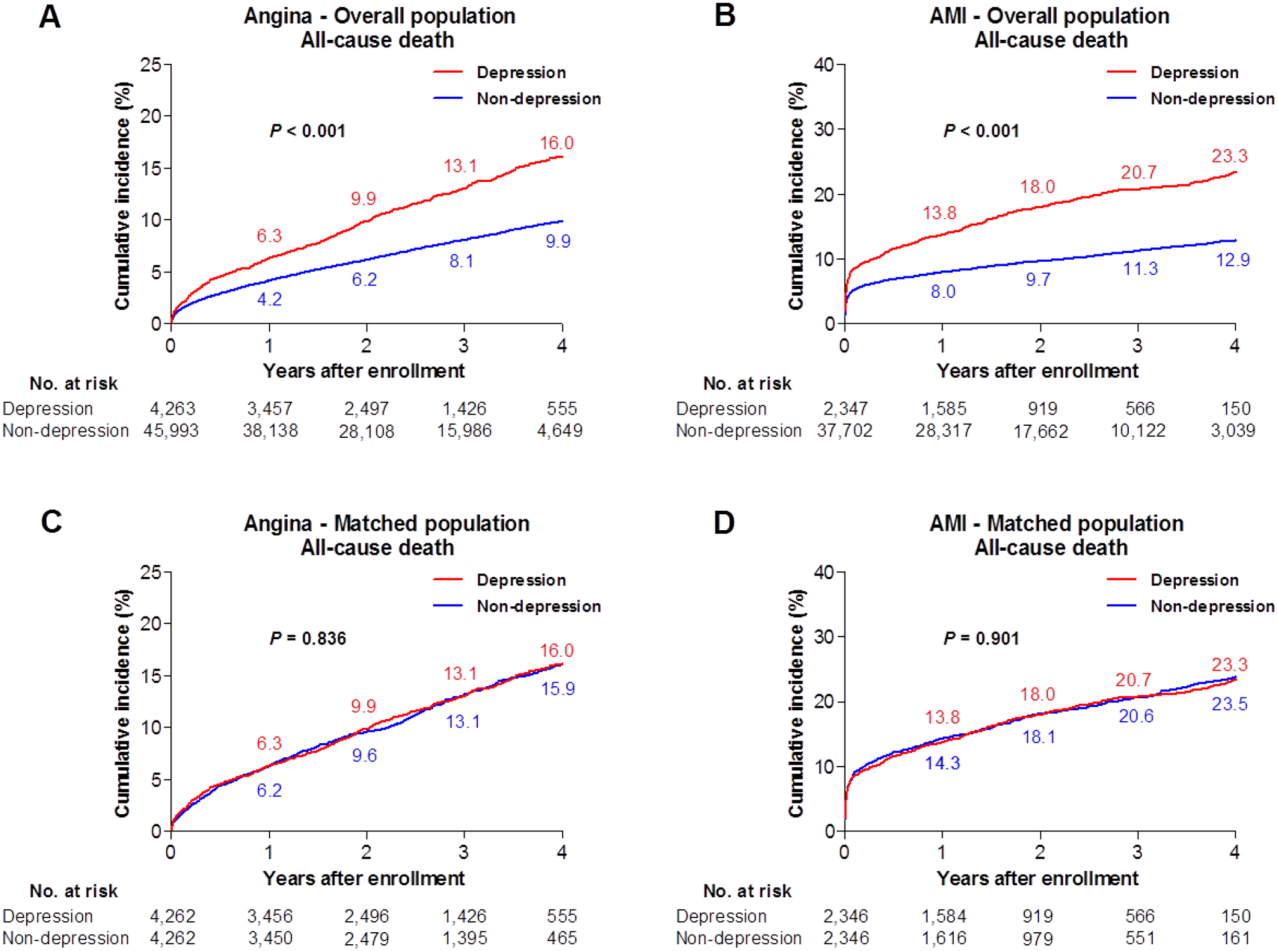
Kaplan–Meier cumulative event curves for mortality between depression and non-depression groups in (**A**) overall angina, (**B**) overall AMI, (**C**) propensity-score matched angina, and (**D**) propensity-score matched AMI populations, respectively. The numbers in each figure represents the cumulative incidence rates at each time point. *AMI* acute myocardial infarction.

Among them, 7.3% of participants (n = 6610) had at least one visit for depression within 6 months before PCI index day (Fig. 1). Table 1 lists the baseline characteristics of the study population according to depressive episodes in the angina and AMI groups. Participants with depression were older, more frequently female, and more frequently had comorbid conditions compared with those without depression in both the angina and AMI groups. In the angina cohort, the prescribing rates of antiplatelet and statin medications were significantly lower in the depression group compared with the non-depression group. In the AMI cohort, the prescribing rate of statin were lower in the depression group compared with the non-depression group (Table 1). Figure 2A,B show the cumulative incidence rates of all-cause death between the non-depression and depression groups (p < 0.001). After propensity score matching, there were 4262 matched pairs of patients in the angina cohort and 2346 matched pairs of patients in the AMI cohort. In the propensity score matched cohorts, there were no differences in demographic or clinical characteristics between subjects with and without depression (Table 2).

**Table 1:** Demographic and clinical profiles of patients according to pre-existing depression.

Baseline characteristics	Angina (n = 50,256)				AMI (n = 40,049)			
	Overall (n = 50,256)	Non-depression (n = 45,993)	Depression (n = 4,263)	p value	Overall (n = 40,049)	Non-depression (n = 37,702)	Depression (n = 2,347)	p value
Age, years	65.4 ± 11.4	65.0 ± 11.5	69.8 ± 9.9	< 0.001	63.6 ± 13.0	63.1 ± 13.0	70.6 ± 11.3	< 0.001
Men	33,912 (67.5%)	31,722 (69.0%)	2,190 (51.4%)	< 0.001	30,239 (75.5%)	28,958 (76.8%)	1,281 (54.6%)	< 0.001
**Cormobidities**								
Diabetes	19,646 (39.1%)	17,470 (38.0%)	2,176 (51.0%)	< 0.001	11,087 (27.7%)	10,073 (26.7%)	1,014 (43.2%)	< 0.001
Hyperlipidemia	25,963 (51.7%)	23,159 (50.4%)	2,804 (65.8%)	< 0.001	11,939 (29.8%)	10,821 (28.7%)	1,118 (47.6%)	< 0.001
Hypertension	33,940 (67.5%)	30,424 (66.1%)	3,516 (82.5%)	< 0.001	19,560 (48.8%)	17,839 (47.3%)	1,721 (73.3%)	< 0.001
Congestive heart failure	4,299 (8.6%)	3,719 (8.1%)	580 (13.6%)	< 0.001	1,419 (3.5%)	1,240 (3.3%)	179 (7.6%)	< 0.001
Arrhythmia	4,572 (9.1%)	4,002 (8.7%)	570 (13.4%)	< 0.001	1,361 (3.4%)	1,213 (3.2%)	148 (6.3%)	< 0.001
Valvular disease	231 (0.5%)	200 (0.4%)	31 (0.7%)	0.012	67 (0.2%)	61 (0.2%)	6 (0.3%)	0.288
Peripheral vascular disease	6,675 (13.3%)	5,769 (12.5%)	906 (21.3%)	< 0.001	3,545 (8.9%)	3,129 (8.3%)	416 (17.7%)	< 0.001
Cerebrovascular disease	7,550 (15.0%)	6,225 (13.5%)	1,325 (31.1%)	< 0.001	3,430 (8.6%)	2,878 (7.6%)	552 (23.5%)	< 0.001
Chronic pulmonary disease	7,765 (15.5%)	6,696 (14.6%)	1,069 (25.1%)	< 0.001	4,454 (11.1%)	3,984 (10.6%)	470 (20.0%)	< 0.001
Moderate to severe liver disease	21 (0.04%)	16 (0.03%)	5 (0.1%)	0.028	15 (0.04%)	13 (0.03%)	2 (0.09%)	0.218
Renal disease	3,002 (6.0%)	2,605 (5.7%)	397 (9.3%)	< 0.001	1,284 (3.2%)	1,110 (2.9%)	174 (7.4%)	< 0.001
Cancer	1,129 (2.2%)	999 (2.2%)	130 (3.0%)	< 0.001	690 (1.7%)	612 (1.6%)	78 (3.3%)	< 0.001
Rheumatologic disease	95 (0.2%)	80 (0.2%)	15 (0.4%)	0.016	53 (0.1%)	47 (0.1%)	6 (0.3%)	0.129
Charlson comorbidity index	1.47 ± 1.46	1.38 ± 1.41	2.38 ± 1.66	< 0.001	0.99 ± 1.27	0.92 ± 1.22	1.96 ± 1.59	< 0.001
**Types of treatment**				< 0.001				0.002
Drug-eluting stent	46,742 (93.0%)	42,786 (93.0%)	3,956 (92.8%)		37,553 (93.8%)	35,379 (93.8%)	2,174 (92.6%)	
Bioresorbable vascular scaffold	318 (0.6%)	310 (0.7%)	8 (0.2%)		251 (0.6%)	244 (0.6%)	7 (0.3%)	
Bare-metal stent	329 (0.7%)	305 (0.7%)	24 (0.6%)		305 (0.8%)	281 (0.7%)	24 (1.0%)	
Balloon angioplasty (no stent)	2,867 (5.7%)	2,592 (5.6%)	275 (6.5%)		1,940 (4.8%)	1,798 (4.8%)	142 (6.1%)	
Number of stent per person	1.42 ± 0.70	1.42 ± 0.70	1.42 ± 0.71	0.892	1.42 ± 0.68	1.42 ± 0.68	1.43 ± 0.67	0.687
**Medication during hospitalization**								
Anti-platelet	49,922 (99.3%)	45,706 (99.4%)	4,216 (98.9%)	0.001	39,923 (99.7%)	37,584 (99.7%)	2,339 (99.7%)	0.706
Statin	44,715 (89.0%)	41,088 (89.3%)	3,627 (85.1%)	< 0.001	37,547 (93.8%)	35,402 (93.9%)	2,145 (91.4%)	< 0.001
B-blocker	29,833 (59.4%)	27,332 (59.4%)	2,501 (58.7%)	0.336	31,428 (78.5%)	29,623 (78.6%)	1,805 (76.9%)	0.059
ACEI/ARB	29,333 (58.4%)	26,862 (58.4%)	2,471 (58.0%)	0.581	27,652 (69.0%)	26,012 (69.0%)	1,640 (69.9%)	0.382

Data are mean ± standard deviation or number (%).

*ACEI* angiotensin-converting enzyme inhibitor, *AMI* acute myocardial infarction, *ARB* angiotensin receptor blocker.

**Table 2:** Characteristics of propensity score matched patients according to pre-existing depression.

Baseline characteristics	Angina (n = 8,524)				AMI (n = 4,692)			
	Overall (n = 8,524)	Non-depression (n = 4,262)	Depression (n = 4,262)	p value	Overall (n = 4,692)	Non-depression (n = 2,346)	Depression (n = 2,346)	p value
Age, years	70.0 ± 10.1	70.1 ± 10.2	69.8 ± 9.9	0.710	70.9 ± 11.3	71.1 ± 11.3	70.6 ± 11.3	0.898
Men	4,327 (50.8%)	2,137 (50.1%)	2,190 (51.4%)	0.999	2,548 (54.3%)	1,267 (54.0%)	1,281 (54.6%)	0.394
**Cormobidities**								
Diabetes	4,370 (51.3%)	2,208 (51.8%)	2,162 (50.7%)	0.548	2,022 (43.1%)	1,014 (43.2%)	1,008 (43.0%)	0.506
Hyperlipidemia	5,589 (65.6%)	2,786 (65.4%)	2,803 (65.8%)	0.464	2,260 (48.2%)	1,143 (48.7%)	1,117 (47.6%)	0.999
Hypertension	7,091 (83.2%)	3,576 (83.9%)	3,515 (82.5%)	0.948	3,466 (73.9%)	1,746 (74.4%)	1,720 (73.3%)	0.841
Congestive heart failure	1,175 (13.8%)	595 (14.0%)	580 (13.6%)	0.700	348 (7.4%)	169 (7.2%)	179 (7.6%)	0.613
Arrhythmia	1,135 (13.3%)	565 (13.3%)	570 (13.4%)	0.744	284 (6.1%)	136 (5.8%)	148 (6.3%)	0.852
Valvular disease	69 (0.8%)	38 (0.9%)	31 (0.7%)	0.810	12 (0.3%)	6 (0.3%)	6 (0.3%)	0.386
Peripheral vascular disease	1,807 (21.2%)	901 (21.1%)	906 (21.3%)	0.700	846 (18.0%)	431 (18.4%)	415 (17.7%)	0.394
Cerebrovascular disease	2,587 (30.3%)	1,263 (29.6%)	1,324 (31.1%)	0.778	1,048 (22.3%)	497 (21.2%)	551 (23.5%)	0.900
Chronic pulmonary disease	2,143 (25.1%)	1,074 (25.2%)	1,069 (25.1%)	0.999	930 (19.8%)	461 (19.7%)	469 (20.0%)	0.680
Moderate to severe liver disease	7 (0.1%)	2 (0.05%)	5 (0.1%)	0.999	5 (0.1%)	3 (0.1%)	2 (0.1%)	0.999
Renal disease	812 (9.5%)	415 (9.7%)	397 (9.3%)	0.910	330 (7.0%)	156 (6.6%)	174 (7.4%)	0.767
Cancer	246 (2.9%)	117 (2.7%)	129 (3.0%)	0.172	145 (3.1%)	68 (2.9%)	77 (3.3%)	0.733
Rheumatologic disease	30 (0.4%)	15 (0.4%)	15 (0.4%)	0.855	10 (0.2%)	4 (0.2%)	6 (0.3%)	0.343
Charlson comorbidity index	2.36 ± 1.67	2.33 ± 1.68	2.38 ± 1.66	0.668	1.90 ± 1.59	1.85 ± 1.61	1.96 ± 1.58	0.730
**Types of treatment**								
Drug-eluting stent	7,905 (92.7%)	3,950 (92.7%)	3,955 (92.8%)	0.800	4,338 (92.5%)	2,165 (92.3%)	2,173 (92.6%)	0.955
Bioresorbable vascular scaffold	11 (0.1%)	3 (0.1%)	8 (0.2%)	0.546	14 (0.3%)	7 (0.3%)	7 (0.3%)	0.999
Bare-metal stent	46 (0.5%)	22 (0.5%)	24 (0.6%)	0.883	48 (1.0%)	24 (1.0%)	24 (1.0%)	0.665
Number of stent per person	1.42 ± 0.70	1.42 ± 0.69	1.42 ± 0.71	0.096	1.43 ± 0.68	1.44 ± 0.69	1.43 ± 0.67	0.465
**Medication during hospitalization**								
Anti-platelet	8,433 (98.9%)	4,218 (99.0%)	4,215 (98.9%)	0.289	4,674 (99.6%)	2,336 (99.6%)	2,338 (99.7%)	0.239
Statin	7,260 (85.2%)	3,634 (85.3%)	3,626 (85.1%)	0.561	4,294 (91.5%)	2,150 (91.6%)	2,144 (91.4%)	0.874
B-blocker	5,016 (58.8%)	2,515 (59.0%)	2,501 (58.7%)	0.774	3,633 (77.4%)	1,829 (78.0%)	1,804 (76.9%)	0.728
ACEI/ARB	4,917 (57.7%)	2,447 (57.4%)	2,470 (58.0%)	0.999	3,283 (70.0%)	1,643 (70.0%)	1,640 (69.9%)	0.704

Data are mean ± standard deviation (SD) or number (%).

*ACEI* angiotensin-converting enzyme inhibitor, *AMI* acute myocardial infarction, *ARB* angiotensin receptor blocker.

### Impact of depressive episodes on all-cause mortality

In the multivariable logistic regression analyses, depression was not associated with in-hospital mortality in either the angina or AMI cohorts. With the multivariable Cox regression analyses, the adjusted incidence of all-cause mortality did not differ between the depression and non-depression groups in the angina cohort (adjusted hazard ratio [aHR] of depression, 1.093; 95% confidence interval [CI] 0.992–1.024; p = 0.074) or the AMI cohort (aHR of depression, 1.107; 95% CI 0.998–1.228; p = 0.054). In the propensity score matched cohorts, there was no significant difference in the incidence of all-cause mortality either in the angina group (hazard ratio [HR] of depression, 1.013; 95% CI 0.893–1.151; p = 0.836) or the AMI group (HR, 0.991; 95% CI 0.865–1.136; p = 0.901). Figure 2C,D show the cumulative incidence rates of all-cause death in the propensity score matched cohort between the Angina (p = 0.836) and AMI groups (p = 0.901). We also found that the subgroup of patients with medicated depression was not associated with all-cause mortality in either the angina group (HR, 1.122; 95% CI 0.975–1.291; p = 0.109) or the AMI group (HR, 1.048; 95% CI 0.900–1.220; p = 0.544) (Table 3).

**Table 3:** Clinical outcomes according to pre-existing depression.

Multivariable analysis	Angina (n = 50,256)		AMI (n = 40,049)	
	Depression with Non-depression		Depression with Non-depression	
In-hospital outcome	Odds ratio (95% confidence interval)	P-value	Odds ratio (95% confidence interval)	P-value
In-hospital mortality	0.999 (0.758–1.318)	0.996	1.100 (0.906–1.335)	0.338
Follow-up outcome	Hazard ratio (95% confidence interval)	P-value	Hazard ratio (95% confidence interval)	P-value
All-cause Death	1.093 (0.992–1.024)	0.074	1.107 (0.998–1.228)	0.054

Propensity score matching analysis	Angina (n = 4,262 pairs)		AMI (n = 2,346 pairs)	
	Depression with Non-depression		Depression with Non-depression	
In-hospital outcome	Odds ratio (95% confidence interval)	P-value	Odds ratio (95% confidence interval)	P-value
In-hospital mortality	1.232 (0.840–1.808)	0.285	1.008 (0.870–1.168)	0.919
Follow-up outcome	Hazard ratio (95% confidence interval)	P-value	Hazard ratio (95% confidence interval)	P-value
All-cause Death	1.013 (0.893–1.151)	0.836	0.991 (0.865–1.136)	0.901

Propensity score matching analysis for AD medicated depression	Angina (n = 3,524 pairs)		AMI (n = 1,868 pairs)	
	Depression with Non-depression		Depression with Non-depression	
In-hospital outcome	Odds ratio (95% confidence interval)	P-value	Odds ratio (95% confidence interval)	P-value
In-hospital mortality	1.000 (0.671–1.490)	0.999	1.208 (0.934–1.563)	0.150
Follow-up outcome	Hazard ratio (95% confidence interval)	P-value	Hazard ratio (95% confidence interval)	P-value
All-cause Death	1.122 (0.975–1.291)	0.109	1.048 (0.900–1.220)	0.544

*AD* Antidepressants, *AMI* acute myocardial infarction.

### Subgroup analyses according to age

Subgroups were analyzed according to age to assess the relative impact of depression on all-cause mortality. In younger matched angina patients (< 65 years), the risk of all-cause mortality was significantly higher in patients with depression compared with those without depression (HR of depression, 1.769; 95% CI 1.240–2.525; p = 0.002), whereas there was no difference in the risk of all-cause mortality in younger matched AMI patients (HR, 1.272; 95% CI 0.848–1.906; p = 0.245). In older matched patients (≥ 65 years), there was no association between all-cause mortality and depression in either the angina (HR, 1.062; 95% CI 0.925–1.221; p = 0.394) or AMI (HR, 1.079; 95% CI 0.933–1.248; p = 0.307) groups. In addition, younger patients with medicated depression in the angina group showed a significantly increased risk of in-hospital mortality (odds ratio of depression, 5.040; 95% CI 1.102–23.063; p = 0.037) and all-cause mortality (HR, 1.592; 95% CI 1.083–2.341; p = 0.018) (Table 4).

**Table 4:** Clinical outcomes according to age.

Univariable analysis	Angina				AMI			
	Age < 65 years (n = 22,956)		Age ≥ 65 years (n = 27,300)		Age < 65 years (n = 21,233)		Age ≥ 65 years (n = 18,816)	
	Depression with Non-depression		Depression with Non-depression		Depression with Non-depression		Depression with Non-depression	
In-hospital outcome	Odds ratio (95% CI)	P-value	Odds ratio (95% CI)	P-value	Odds ratio (95% CI)	P-value	Odds ratio (95% CI)	P-value
In-hospital mortality	2.769 (1.505–5.094)	0.001	0.966 (0.712–1.311)	0.824	1.265 (0.774–2.067)	0.349	1.277 (1.069–1.525)	0.007
Follow-up outcome	Hazard ratio (95% CI)	P-value	Hazard ratio (95% CI)	P-value	Hazard ratio (95% CI)	P-value	Hazard ratio (95% CI)	P-value
All-cause Death	2.614 (2.066–3.308)	< 0.001	1.184 (1.067–1.315)	0.002	1.935 (1.455–2.574)	< 0.001	1.310 (1.174–1.461)	< 0.001

Propensity score matching analysis	Angina				AMI			
	**Age < 65 years (n = 2,368)**		**Age ≥ 65 years (n = 6,152)**		**Age < 65 years (n = 1,288)**		**Age ≥ 65 years (n = 3,394)**	
	**Depression with Non-depression**		**Depression with Non-depression**		**Depression with Non-depression**		**Depression with Non-depression**	
Clinical outcomes	Odds ratio (95% CI)	P-value	Odds ratio (95% CI)	P-value	Odds ratio (95% CI)	P-value	Odds ratio (95% CI)	P-value
In-hospital mortality	2.010 (0.752–5.374)	0.164	0.822 (0.557–1.213)	0.323	1.719 (0.781–3.783)	0.178	1.131 (0.887–1.443)	0.322
Follow-up outcome	Hazard ratio (95% CI)	P-value	Hazard ratio (95% CI)	P-value	Hazard ratio (95% CI)	P-value	Hazard ratio (95% CI)	P-value
All-cause Death	1.769 (1.240–2.525)	0.002	1.062 (0.925–1.221)	0.394	1.272 (0.848–1.906)	0.245	1.079 (0.933–1.248)	0.307

Propensity score matching analysis for AD medicated depression	Angina				AMI			
	Age < 65 years (n = 2,006)		Age ≥ 65 years (n = 5,042)		Age < 65 years (n = 1,050)		Age ≥ 65 years (n = 2672)	
	Depression with Non-depression		Depression with Non-depression		Depression with Non-depression		Depression with Non-depression	
Clinical outcomes	Odds ratio (95% CI)	P-value	Odds ratio (95% CI)	P-value	Odds ratio (95% CI)	P-value	Odds ratio (95% CI)	P-value
In-hospital mortality	5.040 (1.102–23.063)	0.037	1.149 (0.723–1.867)	0.556	1.158 (0.546–2.458)	0.702	1.098 (0.840–1.437)	0.494
Follow-up outcome	Hazard ratio (95% CI)	P-value	Hazard ratio (95% CI)	P-value	Hazard ratio (95% CI)	P-value	Hazard ratio (95% CI)	P-value
All-cause Death	1.592 (1.083–2.341)	0.018	1.111 (0.953–1.295)	0.178	1.412 (0.891–2.237)	0.142	1.178 (0.998–1.390)	0.053

*AD* antidepressansts, *AMI* acute myocardial infarction, *CI* confidence interval.

## Discussion

The present nationwide cohort study investigated the impact of pre-existing depression on mortality in patients with established CAD undergoing PCI. Our study showed that pre-existing depression was not associated with in-hospital mortality or all-cause death in either the angina or AMI groups during a median follow-up period of 2.2 years. However, for patients with angina less than 65 years of age, patients with pre-existing depression had 1.77-times higher all-cause mortality compared with patients without depression. Among them, younger patients with angina who took anti-depressants also showed higher in-hospital mortality and all-cause mortality rates. In the AMI cohort, pre-existing depression was not associated with all-cause mortality.

Our study was conducted using recent NHI claims data on more than 90,000 participants from 2013 to 2017. The prevalence of depression differed depending on the criteria defined by the authors^10^. In our population, the prevalence of depression was 7.3%. In Korean epidemiologic data, the prevalence of major depressive disorder was 4.2–9.1%, which is similar to that of the present study^11^. Furthermore, in case–control studies or studies conducted with self-questionnaires, the prevalence of depression may be affected by several factors, such as recall bias, dementia status, physical function, and marital status. Accordingly, the current study used well-controlled and reliable NHI claims data to alleviate these factors^12,13^. In addition, considering recent nationwide situations in clinical practice and lack of data to determine the relationship between depression and all-cause mortality, the present study was designed.

In 1992, Berkman et al. reported that pre-admission depression was not associated with mortality from myocardial infarction within 6 months^14^. However, the study had a small sample size and selectively included patients from certain hospitals. In 2009, Abrams et al. evaluated the effects of pre-existing and in-hospital psychiatric comorbidities, including depression, anxiety, posttraumatic stress, bipolar, and psychotic disorders, on prognosis after AMI. One-year mortality was significantly associated with pre-existing psychiatric comorbidity, but not with in-hospital psychiatric comorbidity^15^. However, this study did not separately analyze prognosis confined to depression. A nationwide cohort study, which included 170,771 patients with first-time myocardial infarction in Denmark between 1995 and 2014 showed that pre-existing depression increased all-cause mortality by 11%. This association was stronger with current antidepressant use by 22%^16^. However, for treatment of CAD, there has been a significant improvement in the design and carrier systems of devices and the development of new drugs. This study did not reflect these recent enhanced properties.

In the current study, after propensity score matching, pre-existing depression was not associated with all-cause mortality in patients with either angina or AMI who underwent PCI. One possibility of these findings is that the effect size of depression on all-cause mortality may not be large enough to detect a statistically significant difference. Another possibility is that depression may be an indirect mortality factor affecting obesity, diabetes, and medical adherence^17,18^. In addition, CAD events are major stressful life events, and these episodes can be traumatic in people not prepared for such events^19^. Considering that CAD events can have a stronger psychological and physiological effect than depression, another possibility is that the effect of depression on mortality may be diluted.

We also observed the impact of depression on mortality in younger patients with angina, but not in patients with AMI. In other words, the severity of CAD may be a more important factor on mortality than pre-existing depression. Since angina is a less severe disease with a longer life expectancy than myocardial infarction^20^, the influence of depression might be more pronounced. A previous epidemiologic study reported that depression is an independent predictor of mortality due to CAD in young individuals^21^. Depression may elicit adverse microvascular responses among younger patients^22^. Furthermore, in younger angina patients treated with antidepressants, the risk of all-cause mortality was less pronounced during the follow-up period, which might suggest the moderation effect by antidepressants. Therefore, since depression is an important risk factor in younger populations, additional medical attention is required in younger patients with co-morbid depression and angina.

Interestingly, in the present study, younger angina patients taking antidepressants before the index PCI day showed higher in-hospital mortality. The Korean and international clinical practice guidelines recommends that non-pharmacological treatments are favored for patients with mild to moderate symptoms^23,24^. Accordingly, the medicated participants are more likely to experience active or severe depression. Our results assume that the impact on the CAD prognosis may vary depending on the severity of depression. We also consider the possibility of cardiac side effects such as QT prolongation or bradycardia for antidepressants^25^. However, similar to previous studies using administrative databases, the present study had no clinical data regarding cardiac side effects. Therefore, these possibilities should be further investigated in large prospective studies.

The strengths of the present study are as follows. First, in most previous studies, the assessment of depression occurred during or within a few weeks after index hospitalization. However, our study evaluated the clinical impact of pre-existing depression before CAD events. Second, strict study participants using the ICD code F32.X-33.X were evaluated, and subgroup analyses were performed by setting criteria for antidepressant use. Third, to assess the effect of depression on mortality in patients with CAD, the current study was conducted with a homogeneous large-scale cohort (i.e., patients with the first episode of CAD undergoing PCI).

Our study also had several limitations. First, the current study was based on administrative data from the HIRA in South Korea. Similar to previous studies using administrative databases, our study lacked clinical patient data and test results. Thus, our findings might be limited by uncertainties in unmeasured confounding variables that may affect patient management^12,13^. Second, in the present study, we evaluated all-cause mortality as the only end point since the exact information on the cause of death or individual cardiovascular events was not available in the HIRA database. However, considering that all-cause mortality is the most unbiased endpoint and given large sample size of current study population, it would be clinically relevant for the purpose of present study. Third, the results do not imply a causal relationship between variables. However, the two variables (depression and mortality) have a clear temporal relationship. Fourth, our study did not evaluate the severity of depression. However, the Denmark nationwide cohort study reported that the severity of depression does not affect all-cause mortality^16^.

In conclusion, pre-existing depression is not associated with in-hospital mortality or all-cause death in patients with CAD undergoing PCI during a median follow-up period of 2.2 years. However, in younger patients with angina, pre-existing depression is associated with higher mortality. These findings should be further investigated and validated through additional studies.

## Methods

### Data sources

In South Korea, the NHI service is a compulsory social insurance service that provides affordable health coverage for the whole Korean population. All healthcare providers are obligated to join the NHI system on a fee-for-service basis. The Health Insurance Review & Assessment (HIRA) service of South Korea is a quasi-governmental organization that systematically evaluates the medical fees reported from healthcare providers to minimize the risk of redundant and unnecessary medical services. Consequently, all NHI claims are reviewed by the HIRA and are systematically classified and recorded in an independent computerized database^26^. Individual diagnoses in the HIRA database are coded by the International Classification of Diseases, 10th Revision (ICD-10). Information about drugs, medical devices, and procedures can be identified by specific codes in the HIRA database^12,26^. Since the claims data of the HIRA were fully anonymized, this study was approved by the local institutional review board of Ulsan University Hospital, Ulsan, Korea (IRB No. UUH 2018-07-008), which waived the requirement for informed consent. All methods were carried out in accordance with International Ethical Guidelines for Epidemiological Studies (2009).

### Study population

Based on the claims database of the HIRA between July 2013 and June 2017, we identified patients aged 18 years and older who underwent PCI (M6551, M6552, M6561-4, M6571, and M6572) as the diagnosis of CAD (ICD-10 codes I20.X–I25.X). Patients with at least 6 months of eligibility prior to index PCI day were selected. Patients were excluded if the HIRA database indicated a previous history of CAD (ICD-10 codes I20.X–I25.X) within 6 months of index PCI hospitalization to selectively include patients with a first diagnosis of CAD. Patients were categorized as either having with acute myocardial infarction (AMI) or angina pectoris, and each diagnostic category was analyzed separately. AMI was defined using discharge diagnosis codes from HIRA databases (ICD-10 codes I21.X–I22.X).

Information about patients diagnosed with depression was obtained from HIRA databases. Depression was defined as depressive episodes (ICD-10 codes, F32.X) and recurrent depressive episodes (F33.X) within 6 months before PCI index hospitalization. We also examined prescribed antidepressants prior to index PCI hospitalization from HIRA databases. Prescribed antidepressants included selective serotonin reuptake inhibitors (citalopram, escitalopram, fluoxetine, fluvoxamine, paroxetine, and sertraline), serotonin–norepinephrine reuptake inhibitors (desvenlafaxine, duloxetine, milnacipran, and venlafaxine), serotonin modulators (trazodone, vortioxetine, and tianeptine), a norepinephrine–dopamine reuptake inhibitor (wellbutrin), a noradrenergic and specific serotonergic antidepressant (mirtazapine), tricyclic and tetracyclic antidepressants (amitriptyline, amoxapine, clomipramine, doxepin, imipramine, and nortriptyline), and monamine oxidase inhibitors (selegiline and moclobemide). In addition, for subpopulation analyses, the medicated depression group was defined as patients diagnosed with depression and prescribed antidepressants within 6 months before PCI index hospitalization.

### Study variables and endpoint

Within 6 months of the index PCI day, individual comorbid conditions were identified using ICD-10 codes, such as diabetes mellitus, hyperlipidemia, hypertension, congestive heart failure, arrhythmia, valvular heart disease, myocardial infarction, peripheral vascular disease, cerebrovascular disease, chronic pulmonary disease, moderate to severe liver disease, renal disease, cancer, and rheumatologic disease^27^. Patients were also considered to have diabetes mellitus, hypertension, and hyperlipidemia if anti-diabetic, anti-hypertensive, and anti-hyperlipidemic drugs were identified from the medication codes in the HIRA database within 6 months of index PCI day^12^. The Charlson comorbidity index was calculated from these comorbidities^27^. In addition, we obtained information about cardiovascular medications, such as antiplatelet agents, statins, angiotensin-converting enzyme inhibitors, angiotensin receptor blocker, and beta-blockers^12^.

The types of PCI were classified from HIRA claims as drug-eluting stents (codes J5083 or J8083), bioresorbable vascular scaffolds (code J5084), bare-metal stents (codes J5231, J5232, or J8231), or non-stent coronary balloon angioplasty if stent codes were not documented.

The clinical endpoint was all-cause mortality, including in-hospital and post-discharge mortality. All-cause mortality was identified by all in- and out-patient HIRA claim records that indicated death. All claims data until December 2017 were used.

### Statistical analysis

We employed statistical analyses in the angina and AMI cohorts separately. We presented baseline characteristics and comorbidities as mean ± standard deviation for continuous variables and counts (%) for categorical variables. We compared patients’ characteristics between the depression and non-depression groups using the t-test or the Chi-squared test according to continuous or categorical variables, respectively. We computed the cumulative incidence rates using the Kaplan–Meier method and compared them between the depression and non-depression groups using the log-rank test. We used the logistic or Cox regression analyses for in-hospital mortality or all-cause mortality to identify any associations between depression status and clinical outcomes. With multivariable analyses, we considered possible adjustment factors, including age, comorbidities, type of PCI treatment, number of stents, medications during hospitalization, and the Charlson comorbidity index. The final multivariable models were derived using the backward variable selection approach. In addition, to reduce the impact of potential confounding factors and treatment selection between the depression and non-depression groups, propensity score matching method was used. Propensity scores were obtained from logistic regression with covariates of age, comorbidities, type and number of stents, medications during hospitalization, and the Charlson comorbidity index. In the propensity score matching, we employed nearest-neighbor matching using a caliper size of 0.2 multiplied by the standard deviation for linearly transformed propensity scores (logit transformation). We evaluated covariate balancing in light of standardized mean differences. All standardized differences in baseline variables were < 0.05 (5%), and we assumed that covariate balancing was achieved. In addition, we conducted the paired t-test or McNemar test for continuous or categorical variables to examine covariate balancing between the two matched groups. In the propensity score matched cohort, the generalized estimating equation or the Cox regression model with a robust standard error were used to accommodate the clustering of matched pairs. Furthermore, the population with medicated depression was also analyzed with the propensity score matching analysis. We also divided the whole population into two groups of younger patients (age < 65 years) and older patients (age ≥ 65 years) and conducted the subgroup analysis according to age groups. All analyses were performed using R software, version 4.0.2 (R Foundation for Statistical Computing, Vienna, Austria; www.r-project.org). The R “MatchIt” package was used for the propensity score matching^11^. A p-value of < 0.05 was considered significant for all two-sided tests.

### Ethical standards

The authors assert that all procedures contributing to this work comply with the ethical standards of the relevant institutional committees on human experimentation. This study was approved by the local institutional review board of Ulsan University Hospital, Ulsan, Korea (IRB No. UUH 2018-07-008).

## Data Availability

The present study analyzed the National Health Insurance (NHI) claims data in South Korea. Data of the NHI claims are accessible to researchers after permission of the Health Insurance Review & Assessment Service (HIRA) in South Korea. Qualified, interested researchers may request access to these data from the HIRA (http://opendata.hira.or.kr/home.do).
